# Systemically delivered adipose stromal vascular fraction mitigates radiation-induced gastrointestinal syndrome by immunomodulating the inflammatory response through a CD11b^+^ cell-dependent mechanism

**DOI:** 10.1186/s13287-023-03562-7

**Published:** 2023-11-13

**Authors:** Lydia Bensemmane, Fabien Milliat, Xavier Treton, Christine Linard

**Affiliations:** 1 PSE-SANTE/SERAMED/LRMed, Institut de Radioprotection et de Sûreté Nucléaire (IRSN), 92260 Fontenay-Aux-Roses, France; 2 Institut Des MICI, 92200 Neuilly, France

**Keywords:** Stromal vascular fraction, Intestine, Irradiation, Monocyte

## Abstract

**Background:**

Stromal vascular fraction (SVF) treatment promoted the regeneration of the intestinal epithelium, limiting lethality in a mouse model of radiation-induced gastrointestinal syndrome (GIS). The SVF has a heterogeneous cell composition; the effects between SVF and the host intestinal immunity are still unknown. The specific role of the different cells contained in the SVF needs to be clarified. Monocytes–macrophages have a crucial role in repair and monocyte recruitment and activation are orchestrated by the chemokine receptors CX3CR1 and CCR2.

**Methods:**

Mice exposed to abdominal radiation (18 Gy) received a single intravenous injection of SVF (2.5 × 10^6^ cells), obtained by enzymatic digestion of inguinal fat tissue, on the day of irradiation. Intestinal immunity and regeneration were evaluated by flow cytometry, RT-PCR and histological analyses.

**Results:**

Using flow cytometry, we showed that SVF treatment modulated intestinal monocyte differentiation at 7 days post-irradiation by very early increasing the CD11b^+^Ly6C^+^CCR2^+^ population in the intestine ileal mucosa and accelerating the phenotype modification to acquire CX3CR1 in order to finally restore the F4/80^+^CX3CR1^+^ macrophage population. In CX3CR1-depleted mice, SVF treatment fails to mature the Ly6C^−^MCHII^+^CX3CR1^+^ population, leading to a macrophage population deficit associated with proinflammatory environment maintenance and defective intestinal repair; this impaired SVF efficiency on survival. Consistent with a CD11b^+^ being involved in SVF-induced intestinal repair, we showed that SVF-depleted CD11b^+^ treatment impaired F4/80^+^CX3CR1^+^macrophage pool restoration and caused loss of anti-inflammatory properties, abrogating stem cell compartment repair and survival.

**Conclusions:**

These data showed that SVF treatment mitigates the GIS-involving immunomodulatory effect. Cooperation between the monocyte in SVF and the host monocyte defining the therapeutic properties of the SVF is necessary to guarantee the effective action of the SVF on the GIS.

## Background

The gastrointestinal (GI) system rapidly develops some proliferating and differentiating cells, which make it one of the most radiosensitive organs. Exposure to high doses of ionizing radiation disrupts the GI system by damaging proliferating stem cells of the crypts, which alters the histology and physiology of the intestine. Acute exposure to ionizing radiation can cause lethal damage to the gastrointestinal tract. To date, there are no Food and Drug Administration-approved agents available to mitigate radiation-induced intestinal injury [1, 2]. The injection of autologous stem/progenitor cells is emerging as a therapeutic option in gastrointestinal syndrome (GIS). Accordingly, we recently reported that the stromal vascular fraction (SVF) obtained from enzymatically digested adipose tissue promoted the regeneration of the intestinal epithelium, limiting lethality in a mouse model of radiation-induced GIS [3]. SVF is a dynamic and heterogeneous cell population that contains mesenchymal-like stem/stromal cells (MSC), endothelial progenitor cells (EPC), pericytes and hematopoietic and immune cells, all of which perform distinct biological functions [4].

At this time, SVF manufacturing processes for clinical use are performed in compliance with good manufacturing practices and well-defined, appropriate and harmonized release acceptance criteria [4]. Historically, MSCs are the cell subset of interest in the regenerative ability of adipose tissue. Their involvement in the biological mechanisms of SVF has been extensively studied in the context of wound healing, angiogenesis and scar remodeling [5]. Except for a few papers reporting the involvement of macrophages in angiogenesis [6, 7], the role of leukocytes and the importance of their presence are not fully clarified. Previous studies have shown the key role played by immune cells like macrophages in crypt regeneration. Indeed, the depletion of host macrophages or the inhibition of macrophage-derived Wnt impaired recovery from radiation injury with loss of ISC, resulting in poor survival [8, 9]. Macrophages play an important role in coordinating signals from gut microbes and injured epithelium, and thereby transmit regenerative signals to intestinal stem cell (ISC) [10]. In intestine, a substantial fraction of macrophage derives from circulating monocytes, which are constantly replenished to maintain the macrophage pool [11]. Monocytes were known to generate a cytokine storm leading to tissue damage, but were also shown to induce regulatory functions during the inflammatory process [12]. In the mouse, inflammatory monocytes are short-lived and express a high level of CCR2 and Ly6C (also called inflammatory or classical monocytes) and exhibit antimicrobial activity. These inflammatory monocytes are precursors of longer-lived patrolling monocytes (also called non-classical or resident monocytes) by undergoing gradual phenotypic and transcriptional changes. In the intestine, incoming monocytes are Ly6C^+^MCHII^−^ cells, differentiating via Ly6C^+^MCHII^+^ intermediates into Ly6C^−^MCHII^+^ monocytes that gain high expression of CX3CR1 [11]. The Ly6C^hi^ classical monocytes are selectively recruited to inflamed tissues in a CCR2-dependent manner, where they produce inflammatory and antimicrobial factors and contribute to local and systemic inflammation [13]. Ly6C^−^CX3CR1^+^ monocytes are present in both resting and inflammatory tissues and are often found attached to or crawling on the luminal wall of the endothelium [14]. Evidence indicates that non-classical monocytes perform a specialized form of immune homeostasis, serving as initiators of acute inflammatory responses and substantially contributing to tissue remodeling [15, 16].

The aim in this work is to understand the involvement of monocytes in the efficiency of SVF treatment in a mice model for the GIS. We showed that mitigation of the irradiation effect was conferred both by the SVF inducing a dynamic of host monocyte phenotype modification and by the SVF-derived monocytes acting as a potential important cell mediating SVF efficacy.

## Material and method

### Mice

Eight- and twelve-week-old male C57BL/6JRj mice were purchased from Janvier (Le Genest Saint Isle, France), and CX3CR1^GFP/GFP^ knockout mice from the Jackson Laboratory have the endogenous locus disrupted by the insertion of sequence encoding green fluorescent protein (GFP), replacing the first 390 bp of the coding exon (exon 2) of the chemokine (C-X3-C motif) receptor 1 (Cx3cr1) gene. 12-week-old C57BL/6JRj mice were used as SVF donors. The C57BL/6-Tg(UBC-GFP)30Scha/J (GFP mice) from Jackson Laboratory were used to explore the cells homing in the intestine. For this study, 196 mice weighing 22–26 g at the beginning of the experiment were housed in the IRSN animal facilities, which are accredited by the French Ministry of Agriculture for performing experiments on rodents. All experiment procedures in this study were approved and performed in accordance with the guidelines of the French Ministry of Agriculture’s Animal Ethics Committee (EC Directive 2010/63/EU and French Decree 2013–118). The use of animals has been approved by an IRSN ethics committee [CEEA 546 Number 81, APAFiS: #20,866-2019052915252839v1, (IRSN project P19-10); and #28,863-20210106163333391v1 (IRSN project P20-06)]. The manuscript reporting adheres to the ARRIVE guidelines (http://www.nc3rs.org.uk/page.asp?id=1357) for the reporting of animal experiments. In this study, the experimental protocol was in line with standard support treatment recommended for patients presenting an acute radiation syndrome [17]. Thus, the antibiotic Avemix (8 g/L) will be combined with all treatments throughout the experimentation process. The animals were housed by four to a cage, with access to food and water ad libitum and with light and dark cycles. Every effort was made to minimize suffering by adding enrichment such as wooden sticks and tunnels. At the reception of the animals, traceability and individual identification of animals were achieved using microchip implants. Animals were randomly distributed in the cages of the different groups. The experimenters were aware during the allocation, the conduct of the experiment, the outcome assessment and the data analysis. All irradiation exposures were performed under gaseous anesthesia with isoflurane (Aerrane, 104 Baxter SA, Lessines, Belgium). At the time of studies, mice were exposed to isoflurane anesthesia and the euthanasia was performed on unconscious mise by cerebral dislocation. To evaluate the treatment using SVF in each experiments using WT mice and CX3CR1^GFP/GFP^ knockout mice and in WT mice received SVF-depleted CD11b, the mice experimental groups were then divided into 3 groups of 6 mice each: control, irradiated and irradiated-treated.

### Irradiation procedure

Mice were irradiated under anesthesia (with a continuous flow of 1.5% isoflurane in oxygen) on a medical linear accelerator (Elekta synergy^®^) delivering 4MVp X-rays (mean photon energy about 1.3 MeV). Reference dosimetry measurements were taken using a 0.125 cc cylindrical ionization chamber calibrated in dose to water in a mouse equivalent tissue phantom placed on a Plexiglas support. A dose rate of about 2.5 Gy/min in dose to water was used. A localized 2 cm larger abdominal irradiation window containing intestine was chosen to limit upper thorax, head and neck, lower and upper extremity exposure. The mice were exposed to 18 Gy as the sublethal optimal irradiation dose for SVF-related effects. The uncertainty of the dose rate measurement was about 5% at *k *= 2.

### Isolation of SVF

Stromal vascular fraction (SVF) cells were isolated from inguinal fat pad adipose tissue samples of 12-week-old C57BL/6JRj mice donors. Briefly, as previously reported [3], the fat pads were excised, finely cut and incubated in a digestion medium containing 0.1% type I collagenase (Sigma-Aldrich, France), 1% penicillin–streptomycin in MEM-α medium by gentle shaking for 20 min at 37 °C. The samples were mechanically disrupted using the gentleMACS Dissociator (Miltenyi Biotec) and then digested again in MEM-α 0.1% type I collagenase medium, followed by mechanical disruption using the gentleMACS Dissociator. The cell suspension was filtered sequentially through 100 and 70 µm cell strainers and centrifuged (400 g, 10 min) to spin down stromal vascular fraction cell pellets. The pellets were resuspended in phosphate-buffered saline (PBS) for i.v. injection. The mice received 2 × 10^6^ cells in 100 µl PBS 4 h post-irradiation.

### Magnetic depletion of SVF Cell Isolates

The depletion experiments used the Miltenyi MACS system (Miltenyi Biotec, France) according to the manufacturer’s instructions. In brief, up to 1 × 10^7^ screened SVF cells were suspended in 90 µl of MACS buffer (PBS, 0.5% BSA 2 mM EDTA) and incubated with 10 µl of anti-mouse CD11b antibody conjugated to iron particles (Miltenyi Biotec) at 4 °C for 10 min. An additional 1 ml of MACS buffer was added to the cell suspension and loaded 0.5 ml at a time onto a Miltenyi Biotec MACS column prewetted with 0.5 ml of MACS buffer within the magnet chamber. The cell-loaded column was gravity drained and then flushed with 0.5 ml of MACS buffer at least 3 times to remove any additional cells. The effluent was collected and considered to be the CD11b^+^-depleted SVF.

### Isolation of mononuclear cells from lamina propria

To isolate, extraintestinal fat tissue was carefully removed and ileum was then flushed of its luminal content with PBS, opened longitudinally and cut into 1 cm pieces on ice. Epithelial cells and were removed by 20 min incubation in predigestion solution: HBSS (without Ca^2+^ and Mg^2+^) containing 5% FBS, 5 mM EDTA, 1 mM DTT by gentle shaking at 37 °C. Then, ileum pieces were vortexed 10 s and filtered through 100 µm cell strainers. Ileum pieces were again incubated in the predigestion solution 20 min by gentle shaking at 37 °C, then vortexed 10 s and filtered through 100 µm cell strainers. Ileum pieces were transferred into the gentleMACS C Tube (Miltenyi Biotec) containing the enzyme mix (Lamina Propria Dissociation Kit mouse, Miltenyi Biotec) and incubated for 30 min at 37 °C under continuous rotation. Then, the isolated cell suspension was obtained by running in gentleMACS Dissociator and filtered through 100 µm cell strainers. Cells were centrifuged for 5 min at 300 g, resuspended in PBS and used for the subsequent analysis.

### Flow cytometric analysis

Flow cytometry was performed using the BD FACSCanto II and analyzed with FlowJo software (Tree Star, Ashland, OR). Dead cells were excluded through Fixable Viability Dye eFluor staining (eBioscience). Non-specific antibody binding was blocked with an anti-CD16/32 (Mouse Fcγ block clone 2.4G2). The cells were incubated for 30 min in a total of 100 µl PBS with conjugated antibodies from BD Biosciences (France): Panel T: anti-CD45 (V500 conjugated; clone 30-F11); anti-CD3 (FITC conjugated; clone 145-2C11); CD4 (PerCP-Cy5.5 conjugated; clone RM4-5); CD8 (PE-Cy7 conjugated; clone 53–6.7). Panel DC: anti-CD45 (V500 conjugated; clone 30-F11); anti-CD11c (PE conjugated; clone HL3); CD11b (BV421 conjugated; clone M1/70); and CD103 (PerCP-Cy5.5 conjugated; clone M290). Panel neutrophil–monocyte–macrophage from lamina propria cells and SVF: anti-CD45 (V500 conjugated; clone 30-F11); CD11b (BV421 conjugated; clone M1/70); anti-Ly6G (PE conjugated, clone 1A8); anti-Ly6C (FITC conjugated for WT mice or PE for CX3CR1^GFP/GFP^ mice; clone AL-21); anti-MCHII (PerCP-Cy5.5 conjugated; clone M5/114.15.2); anti-F4/80 (Alexa Fluor 647 conjugated; clone T45-2342); from Biolegend (France) anti-CX3CR1 (PE-Cy7 conjugated; clone SA011F11) and anti-CCR2 (Alexa Fluor 647 conjugated; clone SA203G11). Panel for intestinal stem cell from BD Biosciences (France): anti-EpCam (FITC conjugated; clone G8.8); anti-CD44 (PE-Cy5.5 conjugated; clone IM7), anti-CD24 (BV421 conjugated; clone M5E2); from eBioscience: anti-CD166 (PE conjugated, clone eBioALC48). Panel MSC from SVF: anti-CD90 (FITC conjugated; clone 30-H12), anti-CD44 (PE-Cy5.5 conjugated; clone IM7), anti-CD34 (Alexa Fluor 647 conjugated; clone RAM34) and anti-CD31 (PE conjugated; clone MEC13.3) for endothelial cells from SVF (BD Biosciences, France).

Isotype-matched antibodies were used for control staining. All antibodies were used at 1:100 dilution. The concentration of cell suspensions was adjusted to 1 × 10^6^ cells per 100 µl.

### Immunohistology

Sections of 5 µm thickness were de-paraffinized and rehydrated. A pretreatment method using heat-induced epitope retrieval was used, and the non-specific binding was blocked with a protein blocker (DakoCytomation, Trappe, France) 30 min at room temperature (RT). For immunofluorescence, the sections were then incubated with the following primary antibodies against: Ki67 (ab15580; Abcam), EpCam (clone G8.8; Biolegend), CD24 (ab64064; Abcam), Lysozyme (ab108508; Abcam), Ly6C (ab15627; Abcam), CX3CR1 (ab8021; Abcam), F4/80 (Ab6640; Abcam), GFP-FITC (Ab6662; Abcam). Co-labeling with antibodies from the same species was performed using Opal™ Multiplex IHC kits (Akoya). After incubation with primary antibodies, slides were washed in PBS-T three times and probed with appropriate fluorescence-conjugated secondary antibodies one hour at room temperature. After three washes in PBS cell, nuclei were counterstained by Vectashield mounting medium with DAPI (Vector Laboratories).

For MPO immunohistochemistry, endogenous peroxidase was blocked with 3% hydrogen peroxide for 10 min and non-specific binding was blocked with the protein blocker. After antigen retrieval, sections were incubated in primary rabbit anti-MPO (ab9535, Abcam). The EnVision^+^ System horseradish peroxidase (HRP) (DakoCytomation) was used as the secondary reagent for all immunostaining sections. The color reaction was developed with the NovaRED™ kit (Vector Laboratories) and counterstained with Mayer's hemalun.

Digital photographs were taken at × 200 magnification (Zeiss Axiolmager microscope), and intestinal sections were examined using Archimed software.

### Real-time PCR analysis

Total RNA was extracted from ileum and spleen cells with the RNeasy Mini kit (Qiagen), and cDNA was prepared with the SuperScript RT Reagent Kit (Applied Biosystems). Real-time PCR was performed on an ABI Prism 7900 Sequence Detection System. All Taqman primers and probes came from Life Technologies (France). The amounts of loaded cDNA were normalized to GAPDH (glyceraldehyde 3-phosphate dehydrogenase) housekeeping gene as an endogenous control. For relative quantification, the comparative threshold cycle (Ct) method was used, and the delta Ct comparison was used to compare gene expression in cells and tissues. Individual data (− delta Ct) were presented to comply with BJP guidelines [18]. Fold induction was calculated as 2^−ΔΔCt^ for the Arg1/Inos ratio.

### Statistics

Mice survival/mortality in different treatment groups was analyzed by Kaplan–Meier statistics as a function of SVF efficiency using GraphPad Prism-8.0 software. Data are expressed as mean ± SEM. We used one-way or two-way analysis of variance (ANOVA) and then a Tukey posttest to determine the significance of differences. *P* values less than 0.05 were considered statistically significant.

## Results

### SVF treatment modulated intestinal leucocyte population

**Fig. 1:**
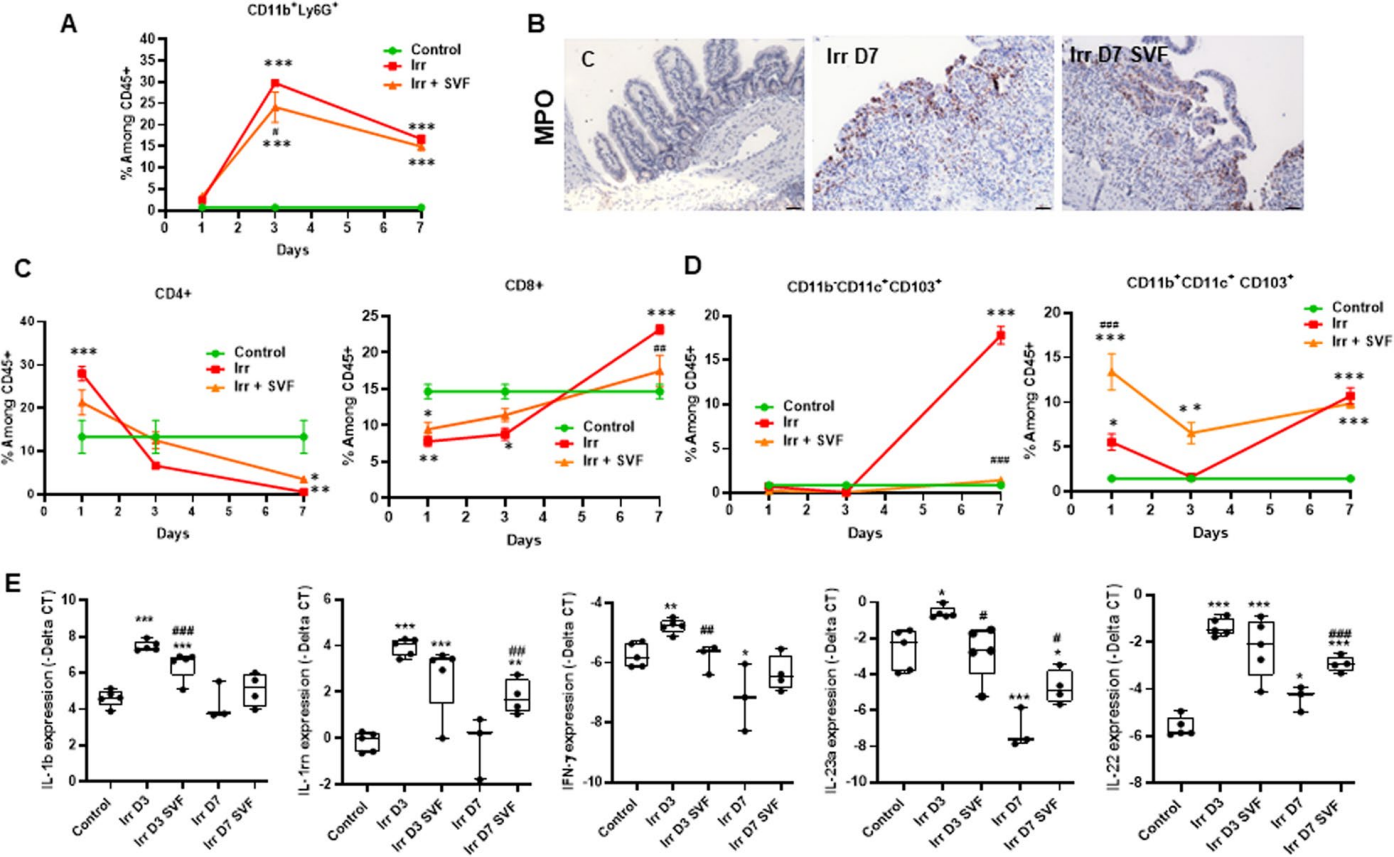
SVF treatment modified intestinal immune cells population after abdominal irradiation. **A** Percentage of neutrophil (CD11b^+^Ly6G^+^) population in ileum of control and at day 1, 3, 7 post-irradiation with or without SVF treatment. **B** Representative MPO immunostaining of the ileum at day 7 post-irradiation with or without SVF treatment. Scale bar: 100 µm. **C** Percentage of CD4^+^ and CD8^+^ population. **D** Percentage of cDC1 (CD11b^−^CD11^+^CD103^+^) and cDC2 (CD11b^+^CD11^+^CD103^+^) population in ileum of control and at day 1, 3, 7 post-irradiation with or without SVF treatment. The data are represented by mean ± SEM (for each groups *n *= 6) **E** Real-time PCR analysis of IL-1β, IL-1Rn, IFN-γ, IL-23a and IL-22 of isolated cell from ileal lamina propria. The comparative threshold cycle (Ct) method was used, and the delta Ct comparison was used to compare gene expression in cells. *P* values were calculated by ANOVA with Tukey correction; *p < 0.05; **p < 0.01; ***p < 0.001 compared with the control group; #*p *< 0.05; ##*p *< 0.01; ###*p *< 0.001 compared with the irradiated group

To investigate the influence of abdominal irradiation on the intestinal immune cell population and SVF treatment, analysis of immune cell population isolated from the lamina propria was performed by flow cytometry on wild-type (WT) mice. Firstly, irradiation induced a significant increase of the neutrophil population at day 3 (43-fold; *p *< 0.001) and day 7 (24-fold; *P *< 0.001) as compared with non-irradiated mice. SVF treatment did not modify the elevation of neutrophil population induced by irradiation at days 3 and 7 (Fig. 1A). Strong neutrophil infiltration was confirmed through staining of myeloperoxidase positive (MPO^+^) cells, mainly localized near the lumen of the intestine, as well as in irradiated mice and in irradiated SVF-treated mice (Fig. 1B). For the CD T helper population, flow cytometry analysis showed a significant increase of the CD4^+^ population and a decrease of the CD8^+^ population from day 1 post-irradiation and inversely a decrease of the CD4^+^ population and an increase of the CD8^+^ population at day 7 post-irradiation. SVF treatment only normalized the CD8^+^ population at day 7 (Fig. 1C).

Next, we analyzed the conventional dendritic cell (cDC) subset population, which is thought to arise from a common DC progenitor, cDC1 (CD11b^−^CD11c^+^CD103^+^) and cDC2 (CD11b^+^CD11c^+^CD103^+^). It is of note that cDC1 were involved in maintaining not only IFN-γ expression from T cells, but also an expression of IFN-γ inducible genes from epithelial cells [19]. SVF treatment normalized the elevation of cDC1 population induced by irradiation at day 7. On the other hand, the cDC2 population is essential for the induction of primary T cell responses in the mucosa [20]. We observed that SVF treatment enhanced significantly the cDC2 population (2.4-fold; *p *< 0.01) from day 1, which remains elevated on day 7 as compared with irradiated mice (Fig. 1D). As cDC2 contributes to Th2-associated responses [20], we analyzed the mRNA level of cytokines from isolated lamina propria cells. Firstly, overexpression of IL-1β and IL-1Rn was observed at 3 days post-irradiation as compared with control mice, which was reduced by SVF treatment (Fig. 1E). Related to Th1 cytokines, overexpression of IFN-γ and IL-23a was observed at 3 days and repression at 7 days in irradiated mice as compared with control mice. SVF treatment normalized their expressions on days 3 and 7.

In addition, IL-22 is produced by T cells and innate lymphoid cells and contributed in both to intestinal barrier function in response to microbiota [21] and to the epithelial and the Transit-Amplifying Cells proliferation [22]. Overexpression of IL-22 occurred at day 3, which did not persist at day 7 in irradiated mice; it is noteworthy that SVF treatment maintains IL-22 expression significantly enhanced at day 7 (*p *< 0.001) compared with irradiated mice (Fig. 1E). Together, these data showed that SVF treatment reduced the inflammatory process and limited the Th1 response.

### SVF treatment modulated intestinal monocyte differentiation

**Fig. 2:**
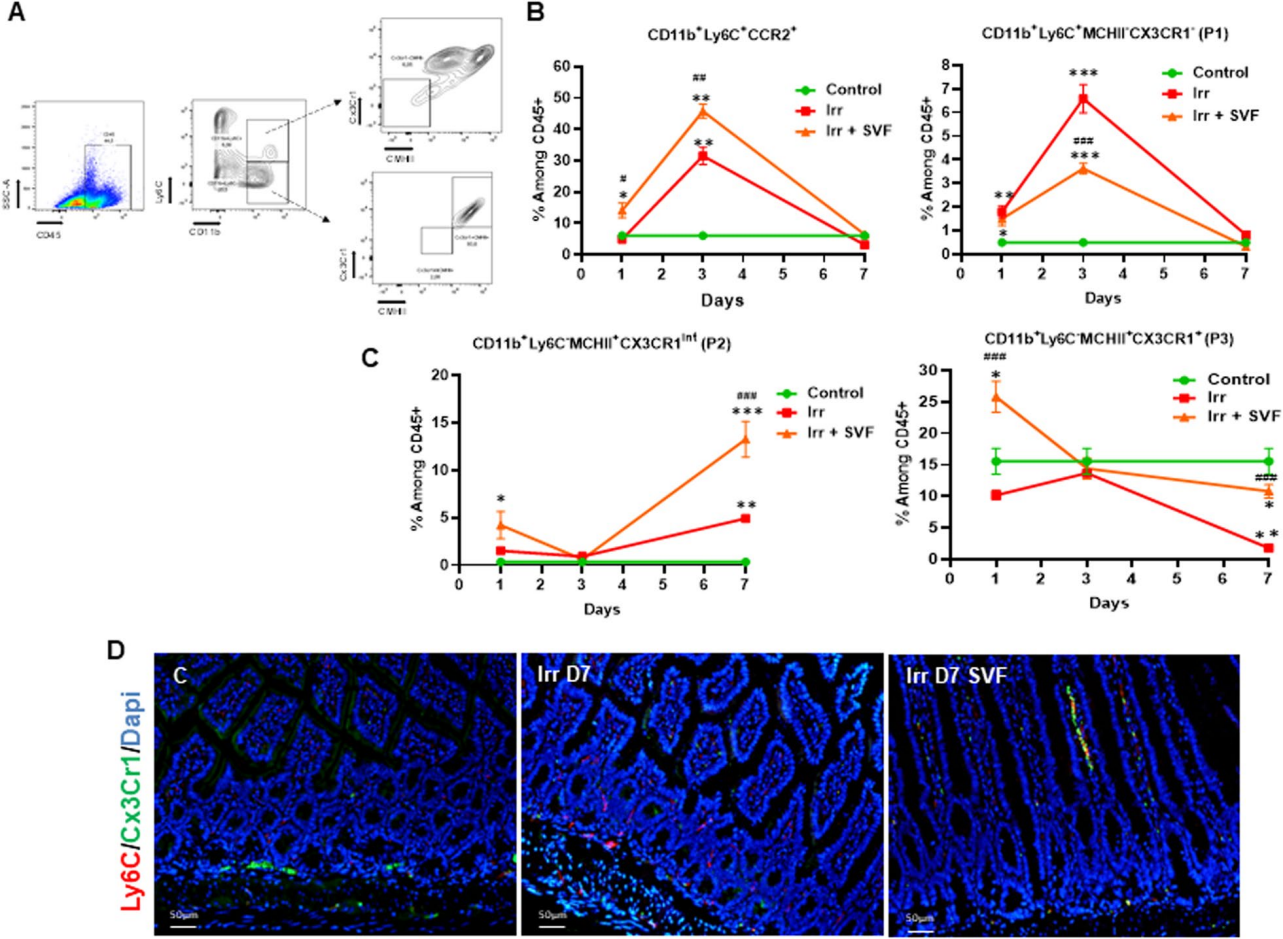
SVF treatment promotes recruitment and maturation of monocytes in ileum. **A** Strategy of monocyte gating. Percentage of **B** CD11b^+^Ly6C^+^CCR2^+^, CD11b^+^Ly6C^+^MCHII^−^ (P1), **C** CD11b^+^Ly6C^−^MCHII^+^CX3Cr1^int^ (P2) and CD11b^+^Ly6C^−^MCHII^+^ CX3Cr1^+^ (P3) among CD45^+^ cells from ileal lamina propria of control and at day 1, 3, 7 post-irradiation with or without SVF treatment. The data are represented by mean ± SEM (for each groups *n *= 6). *P* values were calculated by ANOVA with Tukey correction; *p < 0.05; **p < 0.01; ***p < 0.001 compared with the control group; #*p *< 0.05; ##*p *< 0.01; ###*p *< 0.001 compared with the irradiated group. **D** Representative immuno co-staining Ly6C (red), CX3CR1 (green) in the ileum at Day 7 post-irradiation with or without SVF treatment. Dapi stained nuclei (blue). Scale bar: 50 µm

As different subsets of monocytes are present during intestinal inflammation [14] and as the monocytes can have a protective role in organ damage, notably by activating T cells and innate lymphoid cells [16, 23], we determined the kinetics of monocyte differentiation after irradiation and in cases where SVF treatment modulated the phenotypic differentiation in the lamina propria. According to previous reports [14], when monocytes enter the tissues from the circulation, they are thought to gradually change their phenotype by losing the CCR2 and can be split into three distinct subpopulations in the small intestine based on expression of Ly6C and MCHII: Ly6C^+^MCHII^−^, Ly6C^+^MCHII^+^ and Ly6C^−^MCHII^+^ cells, which can be further divided based on expression of CX3CR1 into CX3CR1^int^ and CX3CR1^+^ cells and definite as CD11b^+^Ly6C^+^MCHII^−^ (P1), Ly6C^−^MCHII^+^CX3CR1^int^ (P2), Ly6C^−^MCHII^+^CX3CR1^+^ (P3). Consistent with this, flow cytometry analysis of the monocyte population isolated from the lamina propria showed that the CD11b^+^Ly6C^+^CCR2^+^ and the P1 populations picked up significantly (5- and 13-fold, respectively) at day 3 post-irradiation as compared with non-irradiated mice (Fig. 2B). SVF treatment increased very early the CD11b^+^Ly6C^+^CCR2^+^ population (15-fold, *p *< 0.05) at 1 day and maintained an increase of both CD11b^+^Ly6C^+^CCR2^+^ and P1 populations at 3 days post-irradiation as compared with non-irradiated mice. The differentiation process occurred at day 7 with a loss of Ly6C; a significant increase of the P2 population (15-fold; *p *< 0.001) was observed in irradiated mice as compared with non-irradiated mice (Fig. 2C). SVF treatment enhanced the P2 population about fourfold and threefold as compared with control and irradiated mice, respectively. When disappearance of the P3 population (*p *< 0.001) was observed at 7 days post-irradiation as compared with non-irradiated mice, SVF treatment restored the percentage of it as compared with non-irradiated mice (Fig. 2C). Double immunostaining of Ly6C/CX3CR1 showed the presence of a few Ly6C^+^ cells and CX3CX1^+^ cells, which are located at the base of the crypts in control mice (Fig. 2D). Seven days post-irradiation, the Ly6C^+^ cells were predominantly present in the ileum. After SVF treatment, an increase in CX3CX1^+^ cells was observed, localized in a chain in the lamina propria.

Together, these results showed that SVF treatment increased the entrance of Ly6C^+^ monocytes into the ileal mucosa and accelerated the modification of the phenotype within 24 h after irradiation, leading to the acquisition of CX3CR1.

### SVF treatment is associated with an accumulation of macrophages in ileum

**Fig. 3:**
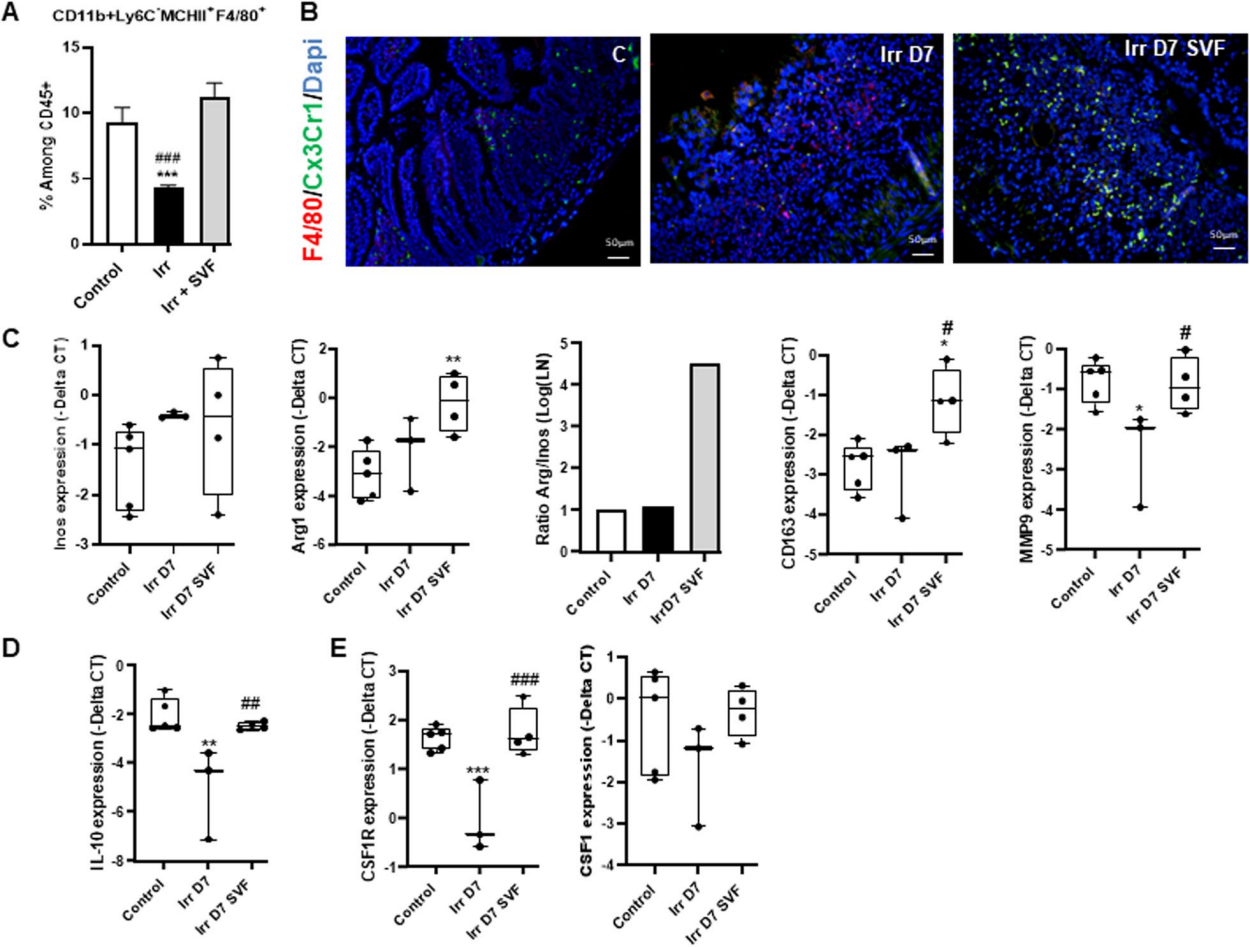
SVF treatment increase the pool of macrophage. **A** Percentage of CD11b^+^Ly6C^−^MCHII^+^ F4/80^+^ among CD45^+^ cells from ileal lamina propria of control and at day 7 post-irradiation with or without SVF treatment. The data are represented by mean ± SEM (for each groups *n *= 6). **B** Representative co-staining of F4/80 (red) and CX3CR1 (green). Dapi stained nuclei (blue). Scale bars: 50 µm. **C**–**E** Real-time PCR analysis of Inos, Arg1, CD163, MMP9, IL-10, CSF1R, CSF1 of isolated cell from ileal lamina propria. The comparative threshold cycle (Ct) method was used, and the delta Ct comparison was used to compare gene expression in cells. *P* values were calculated by ANOVA with Tukey correction; *p < 0.05; **p < 0.01; ***p < 0.001 compared with the control group; #*p *< 0.05; ##*p *< 0.01; ###*p *< 0.001 compared with the irradiated group

Monocyte differentiation into intestinal macrophages involves phenotypic changes with respect to Ly6C, MCHII and CX3CR1 expression, which occurred over 96 h after monocyte infiltration [11]. In this way, flow cytometry analysis of the isolated lamina propria cell population at 7 days post-irradiation showed that the CD11b^+^Ly6C^−^MCHII^+^F4/80^+^ population remains at a very low level (53% fall) in irradiated mice as compared with non-irradiated mice and that SVF treatment normalized this population at 7 days (Fig. 3A). To confirm the presence of macrophage, double immunostaining of F4/80 and CX3CX1 was performed and revealed the rare presence of double staining in irradiated mice; conversely, the predominant presence of F4/80-CX3CX1 double staining cells was observed after SVF treatment (Fig. 3B). As these F4/80^+^CX3CR1^+^ cells adopted an anti-inflammatory phenotype and produced IL-10 [24] and IL-10 is required for maintaining the tolerogenic capability of CX3CR1^+^ macrophages [25], a real-time quantitative RT-PCR was performed on the isolated lamina propria cell population. Firstly, the Arg1/Inos ratio, an indicator of M2/M1 prevalence, revealed M2 predominance induced by SVF treatment, which was confirmed by the CD163 overexpression (fourfold, *P *< 0.05) and MMP9 (3.3-fold, *P *< 0.05) as compared with irradiated mice (Fig. 3C). In addition, SVF treatment restored the level of IL-10 expression (Fig. 3D), suggesting that SVF treatment promoted the induction of a M2-like macrophage.

The proliferation, differentiation and survival of most resident macrophage populations depends upon signals from the macrophage colony-stimulating factor receptor (Csf1R) initiated by Csf1 [26]. On this basis, we analyzed Csf1R expression for the isolated lamina propria cell population and showed that SVF treatment enhanced (twofold) and normalized the mRNA levels of Csf1R as compared with irradiated and control mice, respectively; this was linked to the restoration of Csf1 expression as compared with control mice (Fig. 3E).

### Abdominal irradiation generates more severe damage in mice lacking the CX3CR1 receptor

**Fig. 4:**
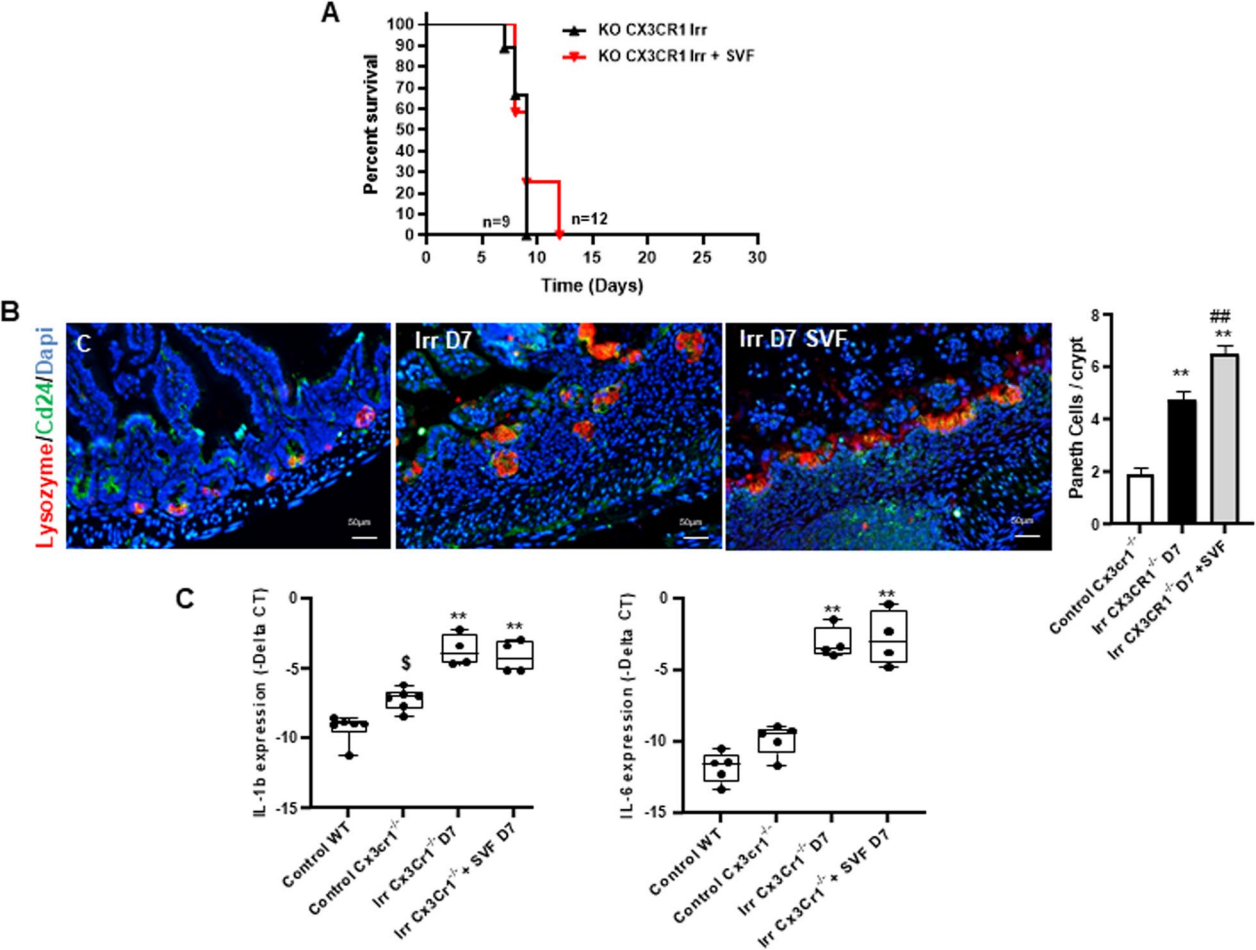
CX3CR1 is required to mitigate irradiation damage by SVF. **A** Kaplan–Meier survival analysis of KO CX3CR1 mice exposed to 18 Gy abdominal irradiation with (*n *= 12) or without SVF treatment (*n *= 9). KO CX3CR1 mice show reduced survival following abdominal irradiation with 100% mortality within 9 days. No significant difference between KO CX3CR1 irradiated and KO CX3CR1 irradiated and treated SVF was observed. **B** IHC staining showing the expression pattern of CD24 (green), Lysozyme (red) in ileum. Dapi stained nuclei (blue). The cross section shows the co-staining of CD24 and lysozyme in the KO CX3CR1 control mice and after irradiation with or without SVF treatment. Scale bars: 50 µm. The histogram shows an increase of the Paneth cell number at day 7 after irradiation with or without SVF treatment in KO CX3CR1 mice. The data are represented by mean ± SEM (for each groups *n *= 4–5). **C** Real-time PCR analysis of IL-1β and IL-6 of ileal tissue. The comparative threshold cycle (Ct) method was used, and the delta Ct comparison was used to compare gene expression in cells. *P* values were calculated by ANOVA with Tukey correction; **p < 0.001 compared with the KO CX3CR1 control group; ##*p *< 0.001 compared with the KO CX3CR1 irradiated group; $*p *< 0.01 compared with the WT control group

It was previously reported that the CX3CR1 receptor is functionally important during sepsis [16], where CX3CR1^+^ macrophages play critical roles in dead cell/pathogen clearance, maintenance of immune surveillance and barrier function [27]. To investigate the role of CX3CR1 in SVF efficacy in GIS, we used the KO (CX3CR1^GFP/GFP^) mice model. We have previously reported that 65% of the irradiated WT mice died within 10 days, and the SVF treatment saved 80% of WT mice from lethality [3]. Kaplan–Meier survival analysis revealed that no KO CX3CR1 mice survived beyond 9 days post-irradiation and that SVF treatment delayed total lethality by only 3 days (Fig. 4A). In the KO CX3CR1 mice, double immunostaining of CD24 (marker of the stem cell compartment) and Lysozyme (marker of Paneth cell) showed that irradiation induced destruction of the ileal architecture, with disappearance of the villi where only rare stem cell compartments persisted at 7 days post-irradiation. SVF treatment did not restore the ileal architecture, but, surprisingly, we observed an abundant rise in Paneth cells in the stem cell compartment, forming Paneth-like cell hyperplasia (Fig. 4B). As Paneth cell hyperplasia is frequently associated with severe inflammation [28], we analyzed the IL-1β and IL-6 expression in the KO CX3CR1 mice. We found that overexpression of IL-1β and IL-6 induced by irradiation was maintained despite SVF treatment (Fig. 4C). Together, these data showed that abdominal irradiation in KO CX3CR1 mice inflicts more severe acute damage and that SVF treatment does not abrogate it.

**Fig. 5:**
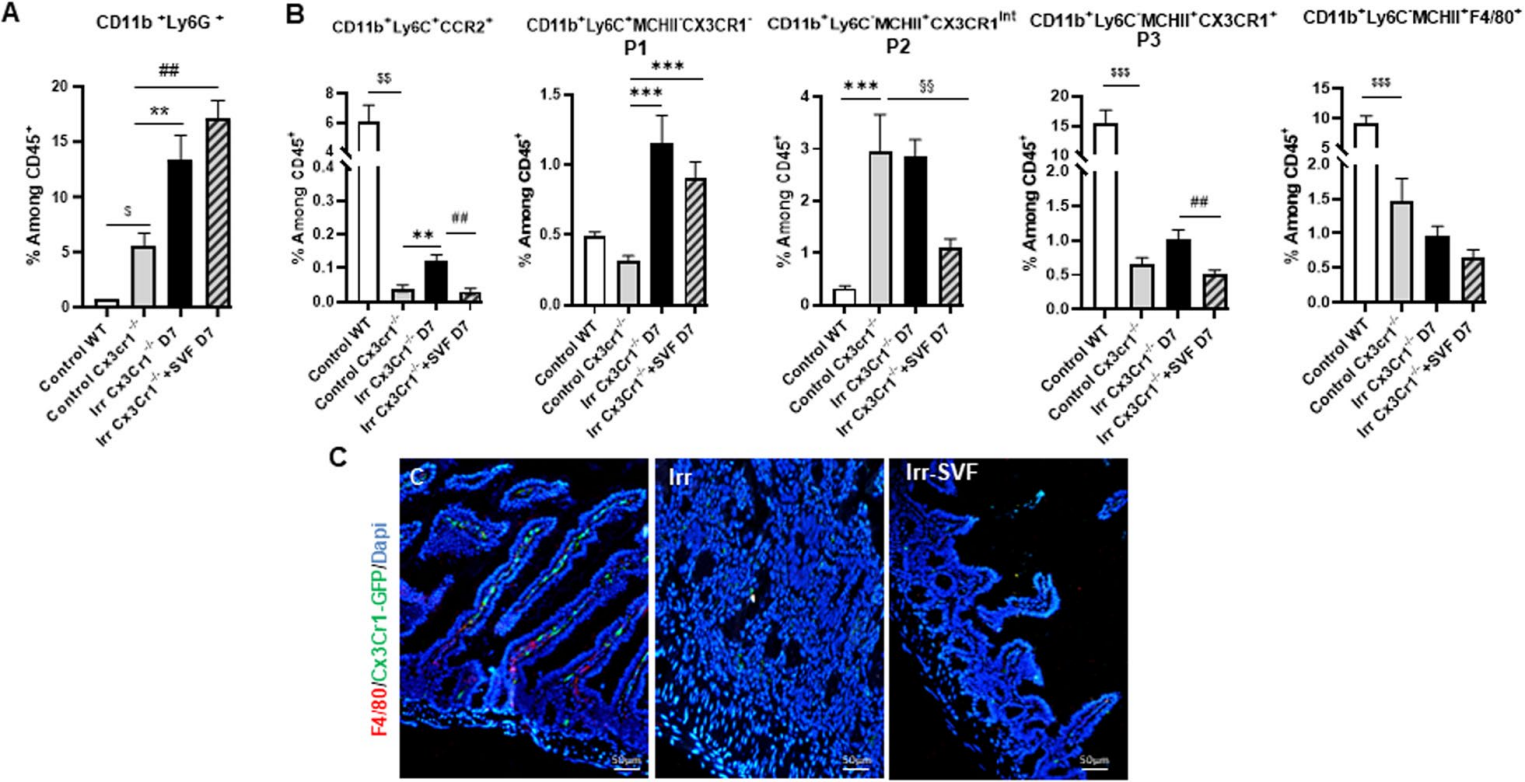
Recruitment and maturation of leucocytes in irradiated KO CX3CR1 mice after SVF treatment. **A** Percentage of neutrophil (CD11b^+^Ly6G^+^) population **B** Percentage of CD11b^+^Ly6C^+^CCR2^+^, CD11b^+^Ly6C^+^MCHII^−^ CX3Cr1^−^ (P1), CD11b^+^Ly6C^−^MCHII^+^CX3Cr1^int^ (P2), CD11b^+^Ly6C^−^MCHII^+^CX3Cr1^+^ (P3) and CD11b^+^Ly6C^−^MCHII^+^ F4/80^+^ among CD45^+^ cells from ileal lamina propria of WT and KO CX3CR1 control and at day 7 post-irradiation with or without SVF treatment in KO CX3CR1 mice (for each groups *n *= 6). **C** Representative immuno co-staining F4/80 (red), CX3CR1-GFP (green) in the ileum at day 7 post-irradiation with or without SVF treatment in KO CX3CR1. Dapi stained nuclei (blue). Scale bar: 50 µm. The data are represented by mean ± SEM (for each groups *n *= 4–5). *P* values were calculated by ANOVA with Tukey correction; *p < 0.01; **p < 0.001 compared with the KO CX3CR1 control group; #*p *< 0.01; ##*p *< 0.001 compared with the KO CX3CR1 irradiated group; $*p *< 0.01; $$*p *< 0.001 compared with the WT control group

Having observed a difference in SVF efficacy for irradiation-induced injury between WT and KO CX3CR1 mice, we characterized the leukocyte subsets recruited in ileum using the flow cytometry strategy detailed before (Fig. 2A). Firstly, we observed a significant rise in the neutrophil population among the CD45^+^ population at steady state in KO CX3CR1 mice compared to WT mice. Irradiation significantly increased the neutrophil population in the KO CX3CR1 mice and SVF treatment maintained this population at a high level (Fig. 5A). Flow cytometry analysis of the monocyte population revealed that the percentage of both Ly6C^+^ and Ly6C^−^ monocytes was impaired in the control KO CX3CR1 mice as compared with the control WT mice (Fig. 5B) as previously observed in other tissue [29]. In KO CX3CR1 mice, irradiation induced an increase of the CD11b^+^Ly6C^+^CCR2^+^ (threefold; *P *< 0.001) and P1 (3.8-fold; *P *< 0.0001) population at 7 days. SVF treatment normalized the CD11b^+^Ly6C^+^CCR2^+^ population, but maintained the percentage of P1 population at a high level. Irradiation did not modify the P2 and P3 populations as compared with control KO CX3CR1 mice and it is of note that SVF treatment did not enhance these 2 populations as compared with irradiated KO CX3CR1 mice. The absence of P3 population maturation by SVF treatment was linked to the low presence of the Ly6C^−^MCHII^+^F4/80^+^ population, which was confirmed by the F4/80^+^ immunostaining (Fig. 5C). Our results showed that the CX3CR1 deficiency impaired both the CD11b^+^Ly6C^+^ and CD11b^+^Ly6C^−^ populations in the intestine. SVF treatment did not enhance the P3 and Ly6C^−^MCHII^+^F4/80^+^ populations, leading to maintenance in a higher proinflammatory environment and intestinal damage. Thus, we have shown that CX3CR1 is crucial to SVF efficiency.

### CD11b-depleted SVF alters GIS mitigation

**Fig. 6:**
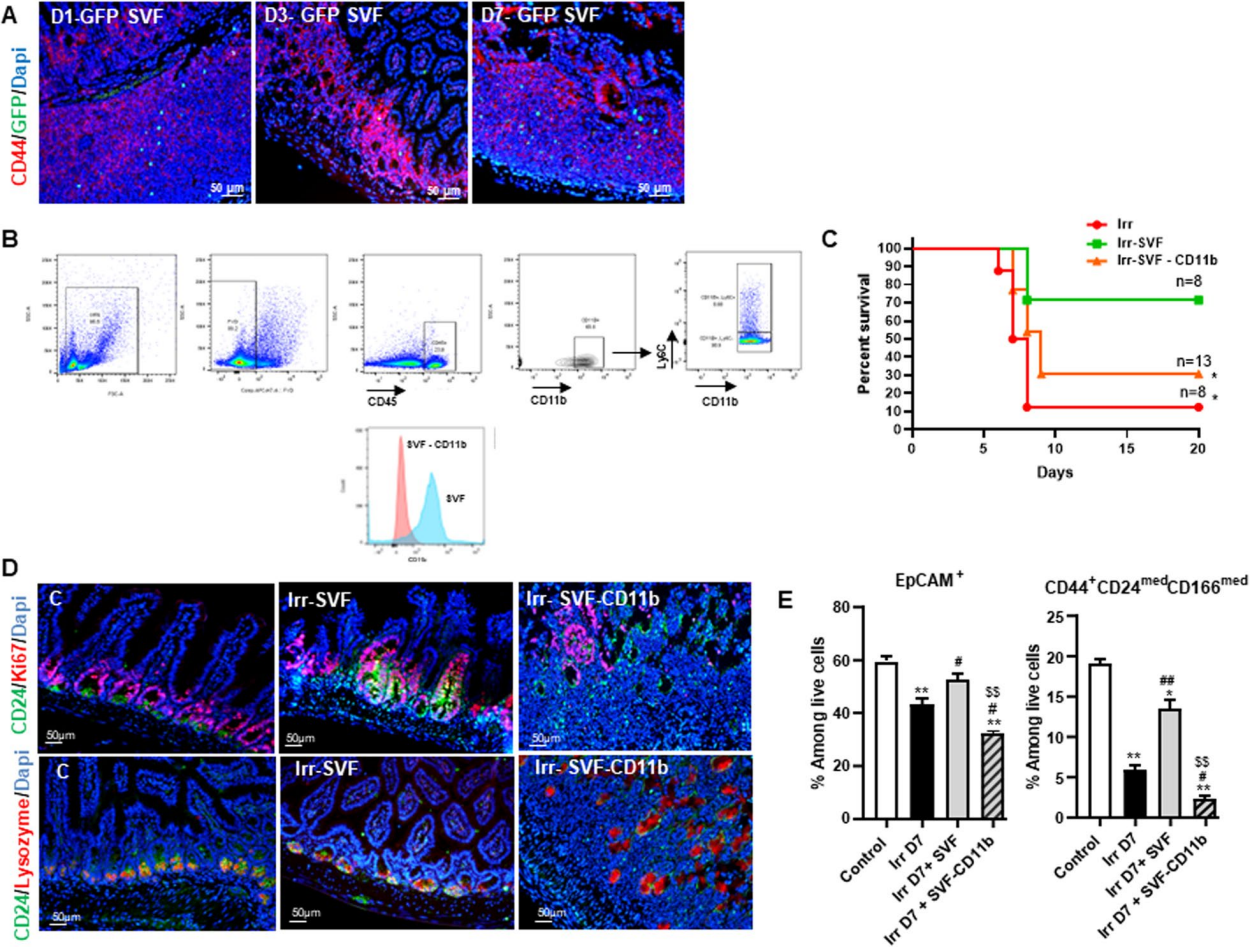
Depletion of CB11b^+^ in SVF alters SVF mitigation. **A** Representative visualization of GFP^+^ SVF cells (green) within ileum from irradiated-GFP-SVF-treated mice at day 1, 3, 7 (for each groups *n *= 6). The stem cell compartment in crypts was identified via CD44 immunostaining (red) and cell nuclei by Dapi (blue) **B** Strategy of CD11b gating and Flow cytometry analysis of SVF-depleted CD11b **C** Kaplan–Meier survival analysis of WT mice WT exposed to 18 Gy abdominal irradiation with or without SVF treatment depleted or not of CD11b, *p < 0.01 Log rank (Mantel–Cox test). **D** IHC co-stained with CD24 (green) and Ki67 (red) to identify the presence of proliferative cells in the intestinal stem cells compartment and IHC co-stained with CD24 (green) and Paneth cell (red) at day 7 post-irradiation of control mice and in irradiated mice with SVF depleted or not of CD11b. Dapi stained nuclei (blue). Scale bars: 50 µm. **E** Flow cytometry analysis of intestinal stem cells compartment (ISC). Flow cytometry analysis of EpCam population identifying the epithelial cell population and CD44, CD24, CD166, combination for ISC population at day 7 after irradiation with or without SVF treatment depleted or not of CD11b. The data are represented by mean ± SEM (for each groups *n *= 5–6). *P* values were calculated by ANOVA with Tukey correction; *p < 0.01; **p < 0.001 compared with the WT control mice; #*p *< 0.01; ##*p *< 0.001 compared with the WT irradiated mice; $$*p *< 0.001 compared with the WT irradiated treated SVF mice

As monocytes/macrophages play a crucial role in the resolution of inflammation and repair and the SVF contains monocytes, we examined whether the presence of monocytes in the SVF are critical for SVF-inducing GIS mitigation. Firstly, injection of the SVF isolated from GFP mice to explore the cells homing in the intestine showed through GFP immunostaining the presence of GFP cells located essentially in the Peyer's patches at day 1 post-irradiation. At day 7, the GFP cells were still present in the Peyer's patches, but were also visible near the base of the crypts (Fig. 6A). Flow cytometry analysis to define the monocyte proportion in our SVF preparation (Fig. 6B) indicated that the SVF contained 73% ± 2% of CD45^−^ cells (including CD90: 10% ± 3%, CD44: 17 ± 3%, CD34: 16% ± 4%, CD31: 18% ± 7%) and 25% ± 2% of CD45^+^ cells. The CD11b^+^ represented 71% ± 7% of the cell population among CD45^+^ cells with 6.9% ± 1.5% of CD11b^+^Ly6C^+^ and 76.0% ± 2.0% of CD11b^+^Ly6C^−^ cells. Firstly, we observed that the treatment with the CD11b-depleted SVF (SVF without CD11b) was lethal in 70% of mice, whereas 70% of mice treated with SVF survived at 20 days post-irradiation (Fig. 6C). To find out whether the SVF without CD11b treatment impacts the proliferation of cells in the stem cell compartment of the ileal crypts, double immunostaining of CD24/Ki67 was carried out. As we have previously shown [3], SVF treatment in irradiated mice increased the number of double CD24/Ki67 positive cells in the crypts, with a large expansion of Ki67^+^ cells in the transit amplification compartment (TA) (Fig. 6D). SVF treatment without CD11b did not induce an increase of the Ki67^+^ cells in the stem cell compartment. Double immunostaining with the CD24/Lysozyme confirmed that the SVF without CD11b did not restore the stem cell compartment. Using flow cytometry, analysis of the epithelial cell population by EpCAM showed that at day 7 post-irradiation the EpCAM^+^ population was significantly decreased (*p *< 0.001) as compared with control mice. The SVF without CD11b treatment did not significantly restore the EpCAM^+^ population as observed in the SVF treatment (Fig. 6E). To confirm the immunostaining analysis of the SVF and the SVF without CD11b treatment on the stem cell compartment, we used the flow cytometry combination previously reported [30] to identify intestinal stem cells (ISCs) with CD44, a select intestinal epithelial crypt cell marker, CD24 and CD166 excluding transient-amplifying and some progenitor cells. A significant decrease of CD44^+^CD166^med^CD24^med^ population (fourfold, *P *< 0.001) was observed at 7 days post-irradiation as compared with control mice. SVF treatment enhanced significantly (threefold; *p *< 0.001) this population as compared with irradiated mice, whereas SVF without CD11b treatment was not effective on this population.

**Fig. 7:**
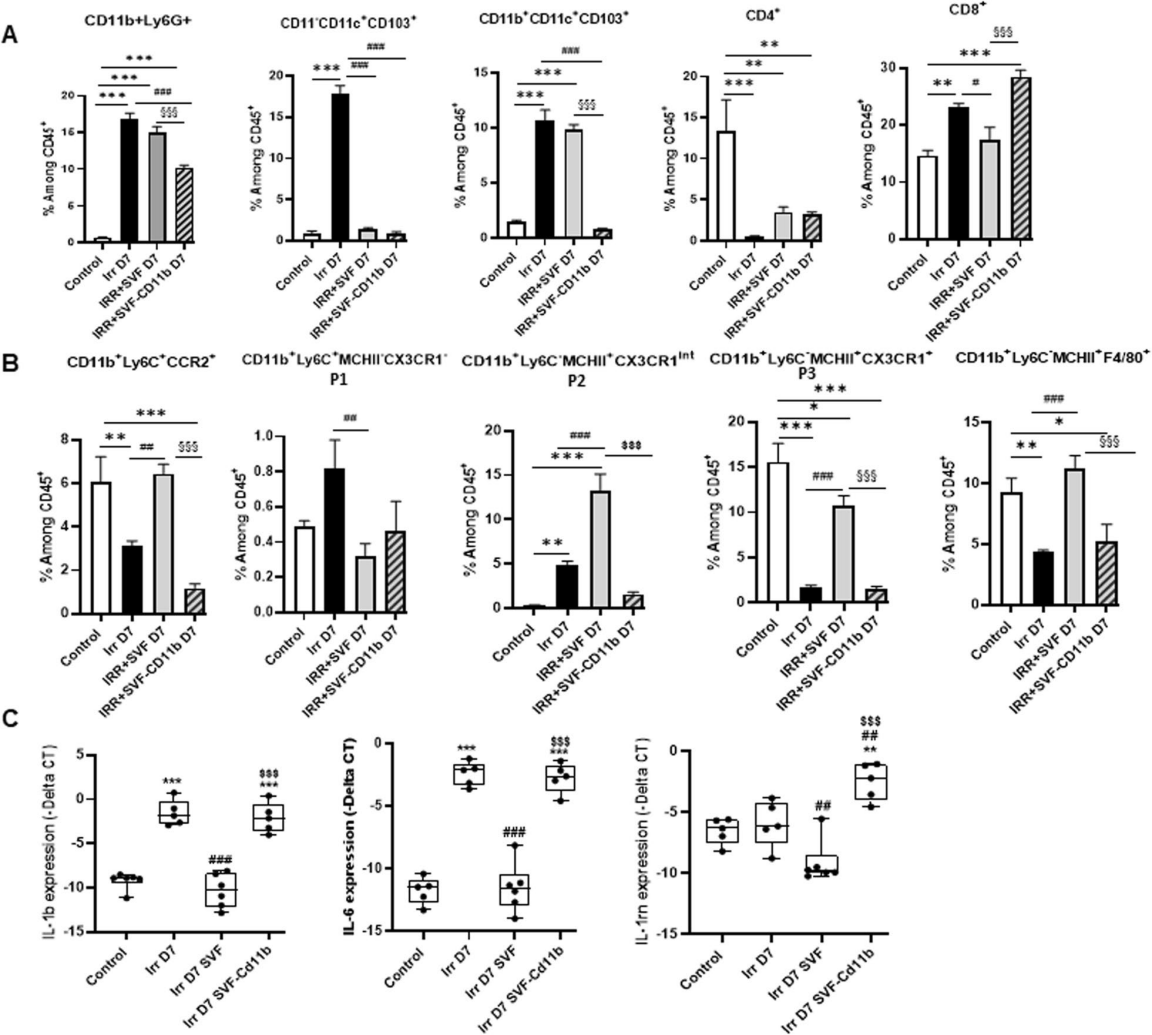
Depletion of CB11b in SVF modifies intestinal immune cells population. **A** neutrophil (CD11b^+^Ly6G^+^), cDC1 (CD11b^−^CD11^+^CD103^+^) and cDC2 (CD11b^+^CD11^+^CD103^+^), CD4^+^ and CD8^+^ population. **B** Percentage of monocytes phenotype CD11b^+^Ly6C^+^CCR2^+^, CD11b^+^Ly6C^+^MCHII^−^ CX3Cr1^−^ (P1), CD11b^+^Ly6C^−^MCHII^+^CX3Cr1^int^ (P2), CD11b^+^Ly6C^−^MCHII^+^CX3Cr1^+^ (P3) and CD11b^+^Ly6C^−^MCHII^+^ F4/80^+^ are analysis among CD45^+^ cells from ileal lamina propria of control and at day 7 after irradiation with or without SVF treatment depleted or not of CD11b. The data are represented by mean ± SEM (for each groups *n *= 6). **C** Real-time PCR analysis of IL-1β, IL-6 and IL-1Rn of ileal tissue. The comparative threshold cycle (Ct) method was used, and the delta Ct comparison was used to compare gene expression in cells. *P* values were calculated by ANOVA with Tukey correction; *p < 0.05; **p < 0.01; ***p < 0.001 compared with the WT control mice; #*p *< 0.05; ##*p *< 0.01; ###*p *< 0.001 compared with the WT irradiated mice; $$$*p *< 0.001 compared with the WT irradiated treated SVF mice

We next examined the effect of SVF without CD11b treatment on immune cell population at day 7 post-irradiation. Unlike SVF treatment, flow cytometry analysis showed that SVF without CD11b significantly decreased the neutrophils and the cDC2 population and enhanced the CD8^+^ induced by irradiation (*P *< 0.001) in the lamina propria (Fig. 7A). Very interestingly, SVF without CD11b treatment reduced significantly the CD11b^+^Ly6C^+^CCR2^+^, P2 and P3 population as compared with SVF treatment, resulting in absence of restoration of the CD11b^+^Ly6C^−^MCHII^+^F4/80^+^ population (Fig. 7B). Real-time PCR analysis revealed the maintaining of the IL-1β and IL-6 overexpression in ileum of SVF without CD11b-treated mice comparable to irradiated mice despite overexpression of IL-1rn (Fig. 7C). Together, these findings showed that in the absence of the CD11b^+^ population in the SVF, the treatment loses its efficiency for stem cell compartment restoration, reducing survival. The presence of the CD11b^+^ population in the SVF enabled an increase of the Ly6C^+^ pool in the intestine, which finally restored the pool of macrophage population in the intestine.

## Discussion

The use of SVF from adipose tissue is safe, easily accessible and compatible with rapid therapeutic countermeasures. Our previous study investigated treatment with SVF to mitigate the GIS [3]. We showed that the intravenous injection of SVF stimulated the regeneration of the epithelium and restored the cell populations in the ISC compartment, increasing survival. Furthermore, due to its heterogeneous cellular composition and the still unknown effects between SVF and the host tissue and the specific role of the cells contained in the SVF, defining the mechanisms of therapeutic efficacy is a challenge. As we previously identified an anti-inflammatory potential of the SVF for the GIS [3], we firstly focused this work on the SVF role in the host immune cell pool and phenotype. Our results unveiled a key role of monocytes in the efficiency of SVF. Unexpectedly, SVF treatment enhanced the number of inflammatory CCR2^+^Ly6C^+^ classical monocytes in the intestine. Previous studies reported the unsuspected role of inflammatory monocytes in controlling inflammation of abdominal disease [12]. Because intestinal resident macrophages were non-proliferative, in inflammatory condition they were continuously replenished from inflammatory CCR2^+^Ly6C^+^ classical bone marrow-derived monocytes; this demonstrates the crucial role of CCR2^+^Ly6C^+^ monocytes for maintaining the adult tissue-resident F4/80^hi^MCHII^hi^ macrophage pool in the intestinal lamina propria [11]. We showed that SVF treatment not only increased the CCR2^+^ monocyte pool in the intestine, but accelerated the monocyte differentiation to Ly6C^−^ non-classical monocytes through a well-known series of intermediaries [11] into proresolving macrophages by downregulating Ly6C, acquiring MCHII expression and upregulating CX3CR1 and finally F4/80 expression. We observed an increase in the presence of P2 and P3 monocytes as early as one day post-irradiation in irradiated SVF-treated mice, while only the P2 population appeared at day 7 in irradiated mice. In a model of Ly6C^+^ monocytes adoptive transfer, the appearance of a Ly6C^−^MCHII^+^CX3CR1^int^ (P2) population was observed at day 2 in the inflamed intestine [14]. This suggests that SVF treatment alleviated rapidly the irradiation-induced blockage of monocyte differentiation into P2 and P3 phenotype. In the inflamed intestine, the maturation process of the P2 population was interrupted from progressing to P3 monocyte by inflammatory signals [11]. The balance shifts more toward an accumulation of Ly6C^int^CX3CR1^int^ cells that retain their pro-inflammatory capacity by the secretion of inflammatory cytokines, including IL-12, IL-23 and IL-1β, thereby promoting type 1 T helper (TH1) and aggravating tissue damage. In this study, we observed that the progression of monocyte differentiation into P3 monocyte promoted by SVF treatment as early as 1 day post-irradiation was linked to down-repression of IL-1β, IL-6, IFN-γ and IL-23. Note that it was very recently suggested that the inflammation does not seem to block monocyte differentiation, but rather drive monocytes to adopt an inflammation-specific expression profile immediately upon entry into tissue [14]. The transcriptional profile of monocytes induced by SVF treatment will be further determined.

The Ly6C^−^MCHII^+^CX3CR1^+^F4/80^+^ macrophage dampened inflammation and promoted tissue repair. As shown by both flow cytometry and immunostaining analysis, SVF treatment restored the macrophage pool in the intestine, which was linked to phenotypical marker expression of M2 macrophages (Arg1, CD163 and MMP9) and upregulation of IL‐10 expression, markers of proresolving macrophages. In the context of an inflamed tissue microenvironment, the inflammatory monocyte population failed or delayed efficient transition to F4/80^+^CX3CR1^+^ macrophages [24], as we observed in irradiated mice. It is noteworthy that macrophages adopt and converse their distinct functional phenotypes in response to the microenvironment. Anti-inflammatory macrophages are known to be induced by Th2-associated responses [31]; at a very early stage, SVF treatment limited the Th1 response, which may also promote the acceleration of macrophage maturation. In addition, CSF-1 signaling is also required for differentiation and maturation of macrophages from monocytes, but also survival of both monocytes and macrophages. A blocking anti-CSF1R antibody induced complete depletion of the macrophage population in the intestine, which disturbs crypt homeostasis with aberrant Paneth cell differentiation and reduction of Lgr5^+^ stem cells. This demonstrated the role of CSF1R-dependent crypt-associated macrophages in the control of stem cell niche maintenance [32]. SVF treatment restored the CSF1R-CSF1 expression, which could also contribute to stem cell compartment restoration. In addition, we have observed that the treatment with SVF without CD11b did not restore the CD44^+^CD166^med^CD24^med^ population. This observation reinforces the importance of the interactions between stem cell compartment and monocytes and the possibility of a role for CSF-1. The even lower CD44^+^CD166^med^CD24^med^ population observed in the SVF without CD11b-treated group compared with the irradiated group may be related to the lower host monocyte recruitment in the SVF without CD11b group; we can hypothesize that the anti-inflammatory effect of MSCs derived from SVF reduces the recruitment of pro-inflammatory monocytes. It was previously shown that CCR2 is required for monocyte migration from the blood into the intestine [14] and CX3CR1 deficiency is linked to increased mortality after LPS injection [27]. Also, the severity of DSS-induced colitis in mice could be limited by the transfer of CX3CR1 macrophages [33]. Importantly, Chousterman et al. [16] showed that CX3CR1 deficiency reduces the adhesive properties of Ly6C^high^ monocytes to endothelium in a kidney damage model during sepsis, demonstrating that the CX3CR1 receptor is involved in monocyte adhesion to the vascular wall; its absence leads to a reduction in inflammatory monocyte adhesion. In this study, using KO CX3CR1 mice, we found that irradiation induced a more severe disease, since 100% lethality was observed at day 9 post-irradiation and SVF treatment had no rescue effect. Particularly, in KO CX3CR1 mice SVF treatment did not limit the inflammatory response or restore the intestinal architecture. Note that the Paneth cell number per crypt remains high in KO CX3CR1 mice after irradiation in the same manner, with and without SVF treatment compared with control mice, while the Paneth cell number was reduced in irradiated WT mice [3]. Paneth cell hyperplasia has been linked to adaptive response of inflamed tissues, to protect the site against infections. However, this response can be negative in KO CX3CR1 mice as these hyperplastic cells contribute to an ill-adapted response to inflammation and in the long term to tumorigenesis through the secretion of growth factors [34]. Our findings showed that in KO CX3CR1 mice, firstly in steady state, the CCR2^+^ monocyte population remained low as compared with WT mice. Although irradiation increased the CCR2^+^ monocyte population in the intestine, Ly6C^−^ differentiation remains blocked; SVF treatment did not abrogate this blockage. These observations strengthen the previous study showing that Ly6C^+^ monocyte recruitment has a beneficial role against damage via a CX3CR1-dependent mechanism [16]. Consistent with this, we showed that CX3CR1 interaction was necessary for SVF efficacy.

Faced with the importance of monocytes in SVF efficiency for GIS and the fact that SVF contains a non-negligible amount of monocytes and macrophages, their influence on GIS mitigation was considered. Our SVF preparation contains a proportion of monocytes similar to human SVF preparation [4] and rodent adipose SVF isolation [35]. It was previously reported that CD14^+^ cells contained in the human SVF induced in vivo a robust angiogenesis where the angiogenesis function induced by SVF CD14^+^ cells was higher than that induced by SVF-derived MSCs [7] and CD11b^+^ cells are required for the vascular function of SVF in inflamed tissue conditions [35], suggesting that the presence of different cell types in the SVF might be beneficial. These observations may confirm the key role of monocytes in repair. Our findings showed that SVF lost its efficiency for survival and anti-inflammatory properties when the CD11b^+^ cells were depleted from the SVF, abrogating stem cell compartment restoration. Our SVF isolation contains about 18% of CD11b^+^ among the total life cells, with a prevalence of Ly6C^−^ monocytes (76%). SVF depletion, in particular of CD11b^+^Ly6C^−^ monocytes, significantly reduced CCR2^+^ monocyte recruitment, which led to defective Ly6C^−^ monocyte maturation, impairing the restoration of the Ly6C^−^MCHII^+^CX3CR1^+^F4/80^+^ macrophage pool. This demonstrated the fundamental role played by CD11b^+^Ly6C^−^ monocytes in SVF efficiency and the interplay between SVF monocytes and host monocyte populations.

Very interestingly, Ly6C^−^CX3CR1^+^ monocytes have been demonstrated to be a source of potent neutrophils activators, facilitating their recruitment [36]. Inversely, different models of neutropenia have provided evidence that monocyte extravasation and macrophage function, notably their antimicrobial activity, depends on neutrophils and their secretion product [37]. An in vivo imaging study revealed that while migrating within microvasculature, monocytes and neutrophils undergo cell–cell contact, whose interactions are increased by inflammation [36]. Here, we observed that in WT mice SVF treatment maintained the neutrophil recruitment induced by irradiation at day 7 in the intestine and, in addition, the decrease of both Ly6C^+^ and Ly6C^−^ monocyte populations seen with SVF without CD11b was linked to a decrease in neutrophil population recruitment. These observations may give rise to the hypothesis that neutrophil–monocyte interactions are probably required for SVF efficiency. In the past, neutrophils have been generally linked to the exacerbation of tissue injury through ROS release, but recent work revealed that neutrophil–monocyte cooperation performs key liver repair functions, with ROS-triggered macrophage skewing toward a reparative phenotype [38]. An essential role of neutrophil was recently reported in a TLR5 mitigation of a lethal acute radiation sickness [39]. In the intestine, excessive neutrophil transmigration created a hypoxic niche in the inflamed area, enhancing epithelial barrier function by increasing the survival of goblet cells, which can form a mucus layer protecting the luminal bacteria [40].

## Conclusion

Earlier in this study, to our knowledge, we reported for the first time that SVF treatment mitigates the GIS-involving immunomodulatory effect. SVF acts at several levels by increasing the CCR2^+^/Ly6C^+^ monocyte pool in the intestine and accelerating its maturation toward a monocyte–macrophage able to promote the anti-inflammatory process and stem cell compartment repair. We assert that cooperation between the monocyte in SVF and host monocyte defining the therapeutic properties of the SVF is necessary to ensure the efficiency of the SVF for the GIS.

## Data Availability

All relevant data and material needed to reproduce the findings are available in the manuscript.

## Abbreviations

SVF: Stromal vascular fraction
GIS: Gastrointestinal syndrome
MSC: Mesenchymal stem cell
ISC: Intestinal stem cell
DC: Dendritic cell
